# Communication Difficulties and their Association with Caregiving Burdens in Aphasic Dementia

**DOI:** 10.1093/geroni/igab046.3572

**Published:** 2021-12-17

**Authors:** Angela Roberts, Haylie Santos, Nathan Gill, Hui Zhang, Elizabeth Salley, Emily Rogalski

**Affiliations:** 1 Northwestern University, Evanston, Illinois, United States; 2 Northwestern University, Chicago, Illinois, United States

## Abstract

Primary progressive aphasia (PPA) is a clinical dementia syndrome for which there is no effective disease-modifying treatment. Impairments in language are the primary and persistent symptoms, and severely limit participation in everyday activities and family conversations. Despite this, there are no published studies examining the objective relationship between conversation difficulties and caregiving burden in PPA. We tested the hypothesis that the severity of care partner perceived conversation difficulties predicts caregiving burden using the Perception of Conversation Difficulty-Dementia Alzheimer’s Type and the Montgomery Borgatta Caregiving Burden Scale. The analysis included baseline data from 78 care partners (62% female) enrolled in the Communication BridgeTM-2 randomized control clinical trial of a speech-language intervention for PPA. Care partners had a mean age of 64.5 years (SD=10.76) and a mean relationship duration with the PPA participant of 38.6 years (SD=15.29). Eighty-six percent were spouses, 5% were adult children, and the remaining 9% were friends or siblings. Higher ratings of conversation difficulties were associated with increased caregiving burden for both objective burden (p < 0.001) and subjective stress burden (p < 0.001). The relationship between conversation difficulties and objective burden was mediated by dependence in activities of daily living and care partner depression, whereas the relationship with subjective stress burden was mediated by depression only. This is the first large scale study of care partner reported conversation difficulties and caregiving burden in PPA. The finding that conversation difficulties have a direct relationship with caregiving burden is an important consideration for interventions and outcome measurement in PPA.

